# ​​Dapagliflozin vs. Empagliflozin for cardiorenal risk reduction: Real-world paired data and comparative study in Indonesia​ 

**DOI:** 10.12688/f1000research.163923.2

**Published:** 2025-08-19

**Authors:** Fonny Cokro, Rani Sauriasari, Dicky Levenus Tahapary, Heri Setiawan, Christian Tricaesario, Nurul Hidayati, Sidartawan Soegondo

**Affiliations:** 1 Department of Clinical and Social Pharmacy, Faculty of Pharmacy, Universitas Indonesia, Depok, West Java, 16424, Indonesia; 2 Department of Pharmacy, School of Medicine and Health Sciences, Atma Jaya Catholic University of Indonesia, Jakarta, Special Capital Region of Jakarta, 14440, Indonesia; 3 Division of Endocrinology, Metabolism, and Diabetes, Department of Internal Medicine, Dr. Cipto Mangunkusumo National Referral Hospital, Faculty of Medicine, Universitas Indonesia, Jakarta, DKI Jakarta, Indonesia; 4 Metabolic, Cardiovascular, and Aging Cluster, The Indonesian Medical Education and Research Institute, Faculty of Medicine, Universitas Indonesia, Jakarta, DKI Jakarta, Indonesia; 5 Department of Pharmacology, Faculty of Pharmacy, Universitas Indonesia, Depok, West Java, 16424, Indonesia; 6 National Metabolomics Collaborative Research Center, Faculty of Pharmacy, Universitas Indonesia, Depok, West Java, 16424, Indonesia; 7 Diabetes Connection and Care, Eka Hospital BSD, South Tangerang, Indonesia; 8 Department of Internal Medicine, Cipto Mangunkusumo National Referral Hospital, Faculty of Medicine, Universitas Indonesia, Jakarta, DKI Jakarta, Indonesia

**Keywords:** Cardiovascular diseases, diabetes mellitus, renal insufficiency, retrospective studies, dapagliflozin, empagliflozin

## Abstract

**Background:**

Previous studies compared the cardiorenal efficacy of these two types of SGLT2 inhibitors; however, the findings are inconsistent and do not reflect the population of Type 2 diabetic Mellitus (T2DM) patients in Indonesia, which ranks fifth globally in the number of diabetic patients. This study aims to evaluate the effects and safety of dapagliflozin and empagliflozin on cardiorenal risk factors in T2DM Indonesian patients over a 12-month.

**Methods:**

This study utilized a multicenter retrospective cohort to evaluate diverse cardiorenal risk factors, encompassing glycemic control, blood pressure, lipid profile, body weight, Body Mass Index (BMI), calculated 10-year Atherosclerotic Cardiovascular Disease (ASCVD) risk, and estimated Glomerular Filtration Rate (eGFR), alongside the safety profile of SGLT2is. Paired data analysis, comparative analysis between groups, and linear regression were conducted to adjust the confounding.

**Results:**

Both groups exhibited enhancements in HbA1c, Fasting Plasma Glucose (FPG), Systolic Blood Pressure (SBP), and Low-Density Lipoprotein Cholesterol (LDL-C). Improvements in BMI, Diastolic Blood Pressure (DBP), triglycerides, ASCVD risk, and High-Density Lipoprotein Cholesterol (HDL-C) were only seen in the dapagliflozin group. The comparative study indicated that dapagliflozin markedly decreased body weight and BMI; however, the results became analogous between groups after correction for confounding variables. No significant differences were observed in the average alteration of HbA1c, FPG, SBP, DBP, LDL-C, HDL-C, triglycerides, total cholesterol, eGFR, and ASCVD risk values. A comparable safety profile was found between groups.

**Conclusion:**

Dapagliflozin and Empagliflozin provide similar advantages in reducing cardiorenal risk and safety after 12 months of treatment in Indonesian patients with T2DM.

## Introduction

Sodium-glucose cotransporter-2 inhibitors (SGLT2is) are now currently the first-line treatment endorsed by worldwide and national guidelines for patients with Type 2 Diabetes Mellitus (T2DM) who have heart failure, Atherosclerotic Cardiovascular Disease (ASCVD), a high-risk of ASCVD, and Chronic Kidney Disease (CKD). ^1– 4^ Apart from that, SGLT2is are also recommended for T2DM patients who are prone to hypoglycemia and are overweight or obese. ^5^ At present, the SGLT2is available in Indonesia are dapagliflozin and empagliflozin, which were launched in 2016 and 2017, respectively. ^6– 9^

Numerous prior extensive retrospective cohort studies have compared the efficacy of empagliflozin and dapagliflozin regarding cardiovascular occurrences, although the findings are inconsistent. ^10– 12^ These studies do not adequately represent the T2DM population in Southeast Asia, especially in Indonesia, which has the fifth-greatest number of T2DM patients globally. ^13^ Diabetes elevates the risk of CKD, coronary artery disease, and heart failure. Cardiorenal complications elevate the risk of mortality, hospital readmission, and deteriorate health-related quality of life. ^14– 16^ Consequently, efficient pharmaceutical approaches to achieve glycemic control and safeguard against cardiorenal complications are essential for patients with diabetes mellitus. Therefore, it is necessary to investigate the actual context of regular clinical practice in Indonesia, acknowledging potential differences in genetics, socioeconomic status, lifestyle, and healthcare systems that may affect cardiorenal outcomes. ^17– 20^ A meta-analysis examining the impact of race or ethnicity on cardiovascular outcomes from antidiabetic treatments indicates that varying racial or ethnic backgrounds may result in disparate outcomes. ^21^ A retrospective cohort study in the United Kingdom showed that white, black, and Asian ethnicities had different glycemic control. Asian patients with T2DM exhibit lower levels of obesity and greater insulin sensitivity compared to Caucasians, ^22^ which may influence cardiorenal outcomes. Thus, this study seeks to address the gap by comparing the effects and safety of dapagliflozin and empagliflozin on cardiorenal risk factors in T2DM Indonesian patients over a 12-month treatment period. The findings will provide valuable insights into these agents’ real-world effectiveness and safety profiles, guiding personalized diabetes management in Indonesia.

## Methods

### Study design, setting and data sources

This research utilized a retrospective cohort. Data gathered from July 2024 to December 2024 came from Rumah Sakit Cipto Mangunkusumo (RSCM) Kencana, SS Diabetes Care, and Diabetes Connection Care (DCC) Eka Hospital in Jakarta and its surroundings. A list of patients who had been using SGLT2i for a minimum of 12 months was obtained from the pharmaceutical installations. Subsequently, adult patients with a history of T2DM were selected in accordance with the inclusion criteria. Data extraction from patient medical records, including both electronic and non-electronic records, was then conducted. All samples were followed up until the end of therapy, transferred to another health facility, or until the end of data collection to evaluate the effectiveness and safety of SGLT2is.

### Study participants

T2DM individuals aged 18 years or older were eligible for participation, as shown by previous medical records or ICD-10 categorization. The sample size calculation was conducted using G*Power software, which determined that a minimum of 210 participants was required for a t-test analysis, based on the assumption of two independent groups, a medium effect size (d = 0.50), a two-tailed distribution, a statistical power of 0.95, and an equal allocation ratio of 1:1.

### Exposure

Participants were categorized into two groups: those receiving dapagliflozin, and those treated with empagliflozin, as monotherapy or combination therapy. Only patients with at least 12 months of SGLT2i use were included to mitigate potential confounding factors. This threshold was based on meta-analysis findings indicating that significant reductions in urine albumin-to-creatinine ratio (UACR) can be observed within 26 to 52 weeks of treatment, ^23^ allowing sufficient time for renal protective effects. Patients with incomplete primary outcome data or inconsistent measurements over 24 months were excluded to minimize bias resulting from non-compliance with therapy adherence.

### Variables

The primary outcome assessed was the change in HbA1c from baseline, serving as an indicator of glycemic control. Secondary outcomes included changes in atherosclerotic cardiovascular disease (ASCVD) risk, assessed via the revised Pooled Cohort Equations (RPCE) calculator ^24^; fasting plasma glucose (FPG); systolic blood pressure (SBP); diastolic blood pressure (DBP); weight; body mass index (BMI); estimated glomerular filtration rate (eGFR); lipid profile; and incidence of adverse drug reactions. Common side effects analyzed included urinary tract infections (UTIs) and genital infections, while severe adverse events such as hypoglycemia, diabetic ketoacidosis, bone fractures, and lower extremity amputations ^25, 26^ were also recorded. Hypoglycemia was defined as random blood glucose levels under 70 mg/dL, ^1, 27^ while UTIs were confirmed via urine or culture tests or documented physician diagnosis. Diabetic ketoacidosis was identified based on ketone presence in blood tests or physician assessment.

Potential confounding factors included age, sex, smoking status, diabetes duration, number of concurrent diabetes medications, and comorbidities such as hypertension, dyslipidemia, cardiovascular disease history, microvascular complications, and adverse drug effects. ^28– 30^ Confounding data were retrospectively extracted from medical records and incorporated into baseline analyses. Missing lipid profile data were imputed using the Sampson-NIH formula, while eGFR was estimated using the CKD-EPI equation. Missing secondary data were handled using linear interpolation for absent baseline values, linear extrapolation for missing intermediate or final data, and mean imputation for non-patterned data.

### Analysis

The Kolmogorov-Smirnov test was used to assess the normality of continuous variables. Parametric data were analyzed using the t-test, while the Mann-Whitney U test was applied for non-parametric variables. The chi-square test was employed to evaluate categorical safety outcomes. A multivariate linear regression model accounted for potential confounders to adjust for baseline differences. Sensitivity analysis was performed by excluding cases with missing data. All statistical analyses were set to have a significance level of p ≤ 0.05 and conducted using SPSS Base Version 22 software.

## Results

**Figure 1.**
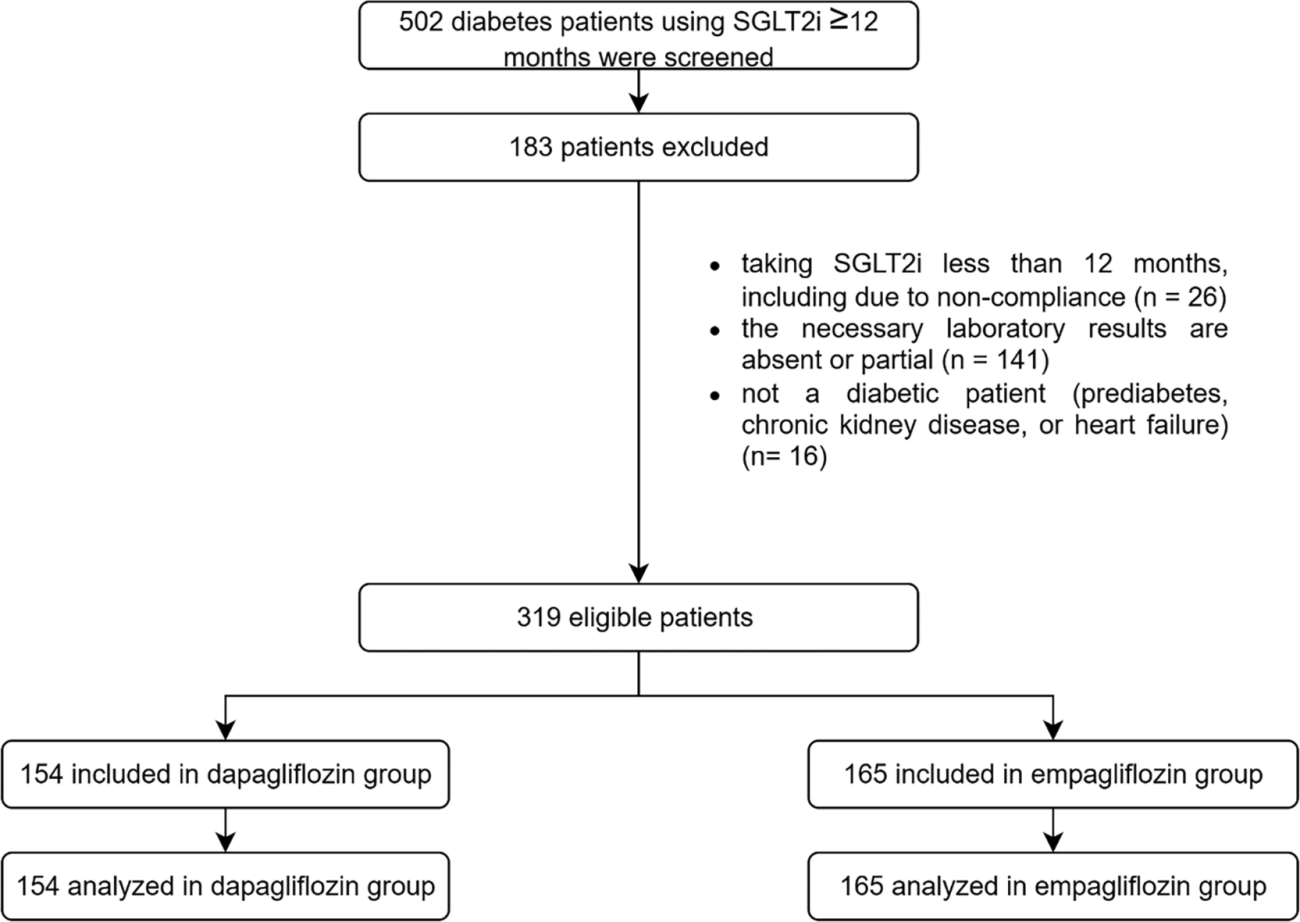
Flowchart of samples. The study was initiated with a screening of 502 patients, and a total of 319 patients were analyzed. The dapagliflozin group consisted of 154 patients, while the empagliflozin group consisted of 165 patients.

A total of 502 eligible patient records were collected from three data-collecting sites, resulting in the inclusion of 319 patients following the screening process, with 154 and 165 patients incorporated and evaluated in the dapagliflozin and empagliflozin groups, respectively, as seen in Figure 1. The baseline demographics exhibited similarities between the groups, except age (55 years in the dapagliflozin group versus 58 years in the empagliflozin group), history of sulfonylurea usage (62 patients in the dapagliflozin group versus 93 patients in the empagliflozin group), history of thiazolidinedione usage (23 patients in the dapagliflozin group versus seven patients in the empagliflozin group), history of acarbose usage (5 patients in the dapagliflozin group versus zero patients in the empagliflozin group), history of beta-blocker usage (10 patients in the dapagliflozin group versus 30 patients in the empagliflozin group), weight (80.7 kg in the dapagliflozin group versus 74.175 kg in the empagliflozin group), and eGFR (90.560 mL/min/1.73 m ^2^ in the dapagliflozin group versus 81.667 mL/min/1.73 m ^2^ in the empagliflozin group), as seen in Table 1. The average duration of SGLT2 is use observed in this study was 14.18 months.

**Table 1.** The baseline demographic of the included samples.

Components	Dapagliflozin (N = 154)	Empagliflozin (N = 165)	p-value
**Age (years)**	55.06 ± 9.940	58.12 ±11.332	0.005
**Gender (male)**	91 (59.091%)	98 (59.394%)	0.956
**Smoking history**	10 (6.493%)	12 (7.273%)	0.784
**Diabetes duration (years)**	3 (2-4)	4 (2-5)	0.243
**Number of diabetes medications**	3 (2-4)	3 (2-4)	0.314
**Medication history:**			
Metformin	131 (85.065%)	127 (76.970%)	0.066
Sulfonylurea	62 (40.260%)	93 (56.364%)	0.004
DPP-4 inhibitor	119 (77.273%)	121 (73.333%)	0.415
GLP-1 agonist	15 (9.740%)	22 (13.333%)	0.317
Metiglinide	0 (0%)	0 (0%)	-
Thiazolidindione	23 (14.935%)	7 (4.242%)	0.001
Acarbose	5 (3.247%)	0 (0%)	0.020
Insulin	40 (25.974%)	36 (21.818%)	0.384
ACE inhibitor/ARB	63 (40.909%)	59 (35.758%)	0.344
Beta-blocker	10 (6.494%)	30 (18.182%)	0.002
Aldosterone antagonist	0 (0%)	1 (0.606%)	1.000
Statin	125 (81.169%)	135 (81.812%	0.881
Aspirin	27 (17.532%)	20 (12.121%)	0.173
**History of illness:**			
Coronary Artery Disease	20 (12.987%)	28 (16.970%)	0.320
Stroke	9 (5.844%)	8 (4.848%)	0.692
Peripheral Artery Disease	6 (3.896%)	6 (3.636%)	0.903
Hypertension	96 (62.338%)	88 (53.333%)	0.104
Dyslipidemia	132 (85.714%)	145 (87.879%)	0.568
Heart failure	3 (1.948%)	4 (2.424%)	0.772
Diabetic nephropathy	30 (19.481%)	27 (16.364%)	0.468
Diabetic neuropathy	12 (7.792%)	16 (9.670%)	0.548
Diabetic retinopathy	5 (3.247%)	7 (4.242%)	0.640
Hypoglycemia	1 (0.649%)	0 (0%)	0.300
HbA1c (%)	8.762 ± 1.776	8.865 ± 1.849	0.612
HbA1c (≤7%)	7 (4.545%)	20 (12.121%)	0.568
Weight (kg)	80.700 ± 15.724	74.175 ± 14.447	0.000
Body Mass Index	29.030 ± 5.639	28.036 ± 4.490	0.054
Fasting Plasma Glucose (mg/dL)	166.182 ± 50.841	165.500 ± 57.081	0.931
Systolic Blood Pressure (mmHg)	133.450 ± 15.806	132.92 ± 17.793	0.801
Diastolic Blood Pressure (mmHg)	78.86 ± 8.260	77.48 ± 9.274	0.130
Low-density lipoprotein Cholesterol (mg/dL)	112.199 ± 42.951	111.156 ± 41.284	0.835
High-density lipoprotein Cholesterol (mg/dL)	43.498 ± 11.756	43.236 ± 10.743	0.622
Triglyceride (mg/dL)	184.048 ± 287.157	180.840 ± 167.420	0.911
Total cholesterol (mg/dL)	193.526 ± 46.950	188.363 ± 55.153	0.437
Estimated Glomerular Filtration Rate (mL/min/1.73 m ^2^)	90.560 ± 26.430	81.667 ± 26.502	0.004
ASCVD Risk (%)	13.526 ± 11.736	13.912 ± 12.703	0.827

Data presented as means (SD), numbers (%), or medians (IQR range) for ordinal data types. DPP-4 inhibitor = Dipeptidyl peptidase-4 inhibitor; GLP-1 agonist = Glucagon-like Peptide-1 Agonist; ACE inhibitor = Angiotensin-Converting Enzyme inhibitor; ARB = Angiotensin Receptor Blocker; ASCVD risk = Atherosclerotic Cardiovascular Disease risk.

Paired data analysis of dapagliflozin and empagliflozin demonstrated a significant reduction in HbA1c (-1.121% vs. -0.986%), FPG (-29.531 mg/dL vs. -25.238 mg/dL), SBP (-8.382 mmHg vs. -4.341 mmHg), and LDL-C (-12.212 mg/dL vs. -17 mg/dL) after 12 months of administration. Nonetheless, substantial decreases in weight, BMI, DBP, triglycerides, ASCVD risk, and a rise in HDL-C were observed exclusively in the dapagliflozin group. Empagliflozin markedly decreased total cholesterol by -16.586 mg/dL. Both groups had a reduction in eGFR at 12 months, which is -0.927 mL/min/1.73 m ^2^ in the dapagliflozin group and -2.169 mL/min/1.73 m ^2^ in the empagliflozin group, as illustrated in Table 2. Both groups had similar patterns in enhancing glycemic control, blood pressure, lipid profile, and ASCVD risk, alongside a reduction in eGFR.

**Table 2.** Effectiveness paired data analysis of Dapagliflozin and Empagliflozin.

Components	n SGLT2is	Means ± SD at baseline	Means ± SD after 12 months	p-value
**Dapagliflozin**				
HbA1c (%)	154	8.762 ± 1.776	7.641 ± 1.189	0.000
Weight (kg)	109	80.879 ± 16.159	78.568 ± 15.186	0.000
BMI	86	29.286 ± 4.831	28.584 ± 4.751	0.000
FPG (mg/dL)	103	164.200 ± 48.210	134.669 ± 38.776	0.000
SBP (mmHg)	109	133.360 ± 16.142	124.978 ± 13.374	0.000
DBP (mmHg)	108	79.030 ± 8.278	76.482 ± 9.689	0.009
LDL-C (mg/dL)	115	112.898 ± 44.024	100.686 ± 32.177	0.042
HDL-C (mg/dL)	85	43.901 ± 11.642	45.303 ± 10.906	0.024
Triglyceride (mg/dL)	92	187.570 ± 318.043	146.901 ± 80.995	0.019
Total Cholesterol (mg/dL)	85	192.114 ± 46.305	177.019 ± 42.454	0.133
eGFR (mL/min/1.73 m ^2^)	97	88.352 ± 26.109	84.819 ± 26.465	0.000
ASCVD Risk (%)	72	12.833 ± 11.657	11.906 ± 12.498	0.044
**Empagliflozin**				
HbA1c (%)	165	8.865 ± 1.849	7.879 ± 1.474	0.000
Weight (kg)	117	74.479 ± 14.665	73.604 ± 14.933	0.118
BMI	103	28.264 ± 4.570	27.948 ± 4.764	0.183
FPG (mg/dL)	136	164.188 ± 55.239	138.950 ± 37.464	0.000
SBP (mmHg)	111	134.100 ± 18.676	129.759 ± 15.514	0.013
DBP (mmHg)	111	77.580 ± 9.696	75.938 ± 10.640	0.187
LDL-C (mg/dL)	143	110.301 ± 40.459	93.301 ± 36.438	0.000
HDL-C (mg/dL)	114	42.973 ± 10.886	43.947 ± 10.999	0.185
Triglyceride (mg/dL)	128	184.367 ± 176.046	168.358 ± 125.796	0.236
Total Cholesterol (mg/dL)	117	187.422 ± 54.937	170.836 ± 46.377	0.005
eGFR (mL/min/1.73 m ^2^)	128	81.063 ± 26.595	78.894 ± 26.706	0.015
ASCVD Risk (%)	81	13.241 ± 12.911	11.388 ± 9.523	0.087

BMI = Body Mass Index; FPG = Fasting Plasma Glucose; SBP = Systolic Blood Pressure; DBP = Diastolic Blood Pressure; LDL = Low-Density Lipoprotein Cholesterol; HDL = High-Density Lipoprotein Cholesterol; eGFR = estimated Glomerular Filtration Rate; ASCVD risk = Atherosclerotic Cardiovascular Disease Risk.

The comparative analysis of effectiveness revealed that dapagliflozin significantly reduced body weight (-2.311 ± 5.173 kg vs. -0.875 ± 4.954) and BMI (-0.702 ± 1.863 vs. -0.316 ± 1.919) compared with empagliflozin. Due to a substantial disparity in baseline weight between the two groups, a supplementary analysis was conducted utilizing the percentage of weight difference, calculated as the difference between initial and final weight divided by the baseline weight, subsequently expressed as a percentage to normalize the baseline discrepancy, yielding robust results that favor dapagliflozin (-2.566% ± 5.863 vs. -1.080% ± 6.493; p = 0.001). No significant changes were seen in the mean values of HbA1c, FPG, SBP, DBP, LDL-C, HDL-C, triglycerides, total cholesterol, eGFR, and ASCVD risk (p > 0.05), as seen in Table 3. Additional analysis to overcome the eGFR difference between the two groups by utilizing the percentage of eGFR difference shows robust results (-3.410 ± 15.343 vs. -2.526 ± 17.913; p = 0.698), meaning no difference between the two groups. The sensitivity analysis, by excluding missing data, yielded a robust conclusion for each comparison. However, the multivariate linear regression results indicated that differences in sulfonylurea use history and baseline body weight between groups influenced the body weight and BMI parameters. The adjusted outcomes for confounding indicated a weight reduction of -1.969 kg in the dapagliflozin group compared to -1.233 kg in the empagliflozin group; p = 0.282 for BMI parameter, and -0.616 in the dapagliflozin group versus -0.367 in the empagliflozin group; p = 0.467.

**Table 3.** Effectiveness comparative analysis of Dapagliflozin vs. Empagliflozin.

Components	Means ± SD Dapagliflozin	Means ± SD Empagliflozin	p-value	N Dapagliflozin	N Empagliflozin
HbA1c Difference (%)	-1.121 ± 1.707	-0.987 ± 1.688	0.482	154	165
Weight Difference (kg)	-2.311 ± 5.173	-0.875 ± 4.954	0.000	109	117
BMI Difference	-0.702 ± 1.863	-0.316 ± 1.919	0.008	86	103
FPG Difference (mg/dL)	-29.531 ± 53.289	-25.238 ± 62.411	0.576	103	136
SBP Difference (mmHg)	-8.380 ± 15.628	-4.340 ± 17.430	0.117	109	111
DBP Difference (mmHg)	-2.546 ± 9.865	-1.638 ± 11.110	0.524	108	111
LDL-C Difference (mg/dL)	-10.779 ± 50.930	0.974 ± 7.809	0.064	116	114
HDL-C Difference (mg/dL)	1.401 ± 10.113	0.974 ± 7.809	0.737	85	114
Triglyceride Difference (mg/dL)	-40.669 ± 280.948	-16.009 ± 165.208	0.415	92	128
Total Cholesterol Difference (mg/dL)	-15.094 ± 56.678	-16.590 ± 62.108	0.861	86	117
eGFR Difference (mL/min/1.73 m ^2^)	-3.533 ± 12.191	-2.791 ± 13.240	0.667	97	128
ASCVD Risk Difference (%)	-0.928 ± 5.409	-1.853 ± 8.212	0.418	72	81

BMI = Body Mass Index; FPG = Fasting Plasma Glucose; SBP = Systolic Blood Pressure; DBP = Diastolic Blood Pressure; LDL = Low-Density Lipoprotein Cholesterol; HDL = High-Density Lipoprotein Cholesterol; eGFR = estimated Glomerular Filtration Rate; ASCVD risk = Atherosclerotic Cardiovascular Disease Risk.

The safety study of both groups revealed no significant differences in any adverse event components, including genital infections, UTI, hypoglycemia, diabetic ketoacidosis, fractures, lower extremity amputations, and overall adverse events (p > 0.05). These results are illustrated in Table 4.

**Table 4.** Safety comparison of Dapagliflozin vs. Empagliflozin.

Safety components	n Dapagliflozin	n Empagliflozin	N Dapagliflozin	N Empagliflozin	p-value
Genital infections	0	0	154	165	
Urinary tract infections	4	5	154	165	1.000
Hypoglycemia	0	1	154	165	1.000
Diabetic ketoacidosis	0	0	154	165	
Fractures	1	1	154	165	1.000
Lower extremities amputation	0	0	154	165	
Any adverse events	5	7	154	165	0.640

## Discussion

According to paired data analysis, dapagliflozin and empagliflozin have comparable efficacy in enhancing glycemic control, specifically for reducing HbA1c and FPG levels. This aligns with the findings of a prior retrospective study including T2DM patients, which demonstrated a reduction in HbA1c in both groups after an average follow-up period of 24.5 months. ^31^ Nonetheless, a separate retrospective trial involving diabetic individuals with a history of chronic kidney disease indicated that dapagliflozin 10 mg was more effective than empagliflozin in reducing HbA1c, at both 10 mg and 25 mg dosages. ^32^

The results of this trial indicate similar efficacy between groups in reducing body weight and BMI; even though before controlling for differences in sulfonylurea history and baseline body weight, the findings favor dapagliflozin. Sulfonylureas augment the visceral fat compartment, resulting in increased body weight ^33^ and biased the body weight outcome. A prior meta-analysis demonstrated a similar result, showing comparable effects between dapagliflozin and empagliflozin on weight gain. ^34^

An extensive retrospective cohort research in Taiwan showed that dapagliflozin resulted in a 31% reduction in LDL-C compared to empagliflozin, particularly in patients with baseline LDL levels below 100 mg/dL. ^11^ The superior reduction in LDL-C within the dapagliflozin group may positively influence the prevention of cardiovascular events. A substantial retrospective cohort research conducted in Korea demonstrated that dapagliflozin administration in patients with T2DM decreased 24% the incidence of cardiovascular mortality and 16% hospitalization resulting from heart failure. ^12^ The findings of the Korean study contradict those of another extensive retrospective cohort trial, which demonstrated empagliflozin’s superiority for both outcomes. ^10^ The disparity is likely due to the later study’s patients having a history of heart failure and being predominantly Caucasian, with only 4.6% identifying as Asian. ^10^ Consequently, dapagliflozin may be more effective in mitigating the risk of heart failure among Asians. The resemblance of ASCVD risk values between the two groups is attributable to the similar risk assessment components in this study, which include comparable SBP, HDL-C, and total cholesterol values. ^35^ The congruence of SBP values is corroborated by prior meta-analyses, indicating no significant difference between dapagliflozin 10 mg and empagliflozin at both 10 mg and 25 mg dosages. ^34^

A prior retrospective cohort study examining kidney function in individuals with T2DM and CKD stages G1-G4 over an 18-month follow-up revealed that the dapagliflozin 10 mg group exhibited a superior increase in eGFR compared to the empagliflozin dosages of 10 and 25 mg. ^32^ A separate retrospective investigation indicated an elevation in eGFR after an average usage length of 23.7 months, based on paired data analysis results, in both the dapagliflozin and empagliflozin groups. ^31^ However, the findings of the prior study contrast with those of the current study, likely attributable to the shorter follow-up period in the latter. Furthermore, the patient features in the present investigation diverge from those in the prior comparative analysis, which exclusively included patients with a history of CKD.

The findings of this investigation indicated comparable safety between the two groups. A retrospective cohort conducted in Turkey demonstrated analogous findings, particularly with the frequency of urinary tract infections and genital infections. ^31^ Furthermore, the utilization of SGLT2is is regarded as safe, presenting a limited risk of adverse events; however, with the increasing risk of genital infections, including in Asian. ^36– 39^ Adverse effects in the current study may remain unrecorded concerning the documentation of safety components. Thus, further evaluation is highly recommended.

This study has the advantage of including patients from various levels of healthcare facilities, from primary to tertiary levels. Nonetheless, multiple limitations exist in the study; primarily, it is a retrospective analysis characterized by missing data. In this case, researchers have implemented multiple data imputation techniques and conducted sensitivity analyses, which yielded robust outcomes. The second constraint is the significant difference between the two groups in some of the baseline characteristics; however, the adjustment has been made to the affected result. The third constraint is the potential for non-compliance, which may impact the outcomes. Last but not least, this study was conducted exclusively in an Indonesian population, which may limit the generalisability of the findings to populations in other geographic regions. Cultural factors, genetic backgrounds, disease epidemiology, and treatment access may differ in other countries. However, the observed associations may still be relevant to similar settings in Southeast Asia or other low- and middle-income countries with comparable patient profiles and healthcare infrastructures.

## Conclusion

Dapagliflozin and empagliflozin both effectively reduced cardiorenal risks in Indonesian T2DM patients. Although dapagliflozin demonstrated additional advantages in terms of weight loss and BMI reduction, these disparities were not statistically significant following the adjustment. Both medications exhibited comparable safety profiles, supporting their use as equally effective options in personalized diabetes care. Longer-duration, larger-scale studies are needed to confirm the results of this study.

## Ethical considerations and consent

Before the commencement of the investigation, the research design underwent evaluation by the Investigation Ethics Committee of RSCM (KET-749/UN2.F1/ETIK/PPM.00.02/2024) dated September 26, 2024; and the Atma Jaya Catholic University of Indonesia (01/05/KEP-FKIKUAJ/2024) dated May 7, 2024. There is no necessity for informed assent in this study, as it is retrospective.

## Data Availability

Figshare: Raw Material. https://doi.org/10.6084/m9.figshare.28748486. ^40^ This project contains the following underlying data:
• Dapa vs. empa Figshare 29042025.xlsx Data are available under the terms of the Creative Commons Attribution 4.0 International license (CC-BY 4.0). Figshare: STROBE checklist. https://doi.org/10.6084/m9.figshare.28747190. ^41^ Data are available under the terms of the Creative Commons Attribution 4.0 International license (CC-BY 4.0). STROBE Checklist for Cohort Studies was employed in the design and reporting of this research. ^42^
