# Supplementary material for: ​​Dapagliflozin vs. Empagliflozin for cardiorenal risk reduction: Real-world paired data and comparative study in Indonesia​ 
Source: F1000Res. 2025 Sep 10;14:569. Originally published 2025 Jun 9. [Version 3] doi: 10.12688/f1000research.163923.3 (PMC12475896; doi:10.12688/f1000research.163923.3)
Supplement: Peer review history

# Reviewer report: Reviewer response for version 3

**DOI:** 10.5256/f1000research.187854.r413133

**Published:** 2025-09-26

**Authors:** FARAZUL HODA

**Affiliations:** 1 Jamia Hamdard University, New Delhi, India

After revision, this article can be accepted.

Is the work clearly and accurately presented and does it cite the current literature?

Yes

If applicable, is the statistical analysis and its interpretation appropriate?

Yes

Are all the source data underlying the results available to ensure full reproducibility?

Yes

Is the study design appropriate and is the work technically sound?

Partly

Are the conclusions drawn adequately supported by the results?

Partly

Are sufficient details of methods and analysis provided to allow replication by others?

Partly

Reviewer Expertise:

Diabetes and Diabetic Kidney Disease

I confirm that I have read this submission and believe that I have an appropriate level of expertise to confirm that it is of an acceptable scientific standard.

# Reviewer report: Reviewer response for version 2

**DOI:** 10.5256/f1000research.186699.r407992

**Published:** 2025-09-05

**Authors:** Aimen Shafiq

**Affiliations:** 1 Dow University of Health Sciences, Karachi, Pakistan

This retrospective cohort study compares the effectiveness and safety of dapagliflozin and empagliflozin over 12 months in Indonesian patients with Type 2 Diabetes Mellitus. Both drugs showed similar improvements in cardiorenal risk factors, with dapagliflozin offering slight advantages in weight and BMI reduction, though these were not significant after adjusting for confounders. Safety profiles were comparable.

 My Comments:

 1) Grammar and awkward phrasing occur frequently, which affect the readability:

- Example: “...which ranks fifth globally in the number of diabetic patients.” This could be rephrased as "Indonesia, which ranks fifth globally in diabetes prevalence."
- “The comparative study indicated that dapagliflozin markedly decreased body weight and BMI; however, the results became analogous between groups...” Consider: "Although dapagliflozin was associated with greater reductions in weight and BMI, these differences were not statistically significant after adjustment."

2) Inconsistent terminology: Use of “SGLT2is” vs. “SGLT2 inhibitors” vs. “SGLT2i” should be standardized.

 3)  It’s unclear whether patients were randomly selected or if clinician discretion influenced who received dapagliflozin vs. empagliflozin. This may introduce treatment allocation bias.

Is the work clearly and accurately presented and does it cite the current literature?

Yes

If applicable, is the statistical analysis and its interpretation appropriate?

I cannot comment. A qualified statistician is required.

Are all the source data underlying the results available to ensure full reproducibility?

Yes

Is the study design appropriate and is the work technically sound?

Yes

Are the conclusions drawn adequately supported by the results?

Yes

Are sufficient details of methods and analysis provided to allow replication by others?

Yes

Reviewer Expertise:

Cardiovascular

I confirm that I have read this submission and believe that I have an appropriate level of expertise to confirm that it is of an acceptable scientific standard.

## References

[1] : Empagliflozin in the treatment of heart failure. *Future Cardiology* .2024;20(5-6) : 10.1080/14796678.2024.2360818 251-261 10.1080/14796678.2024.2360818 PMC1131872538865086

[2] : The dual role of empagliflozin: Cardio renal protection in T2DM patients. *Annals of Medicine & Surgery* .2022;81: 10.1016/j.amsu.2022.104555 10.1016/j.amsu.2022.104555 PMC948686236147179

# Response

**Published:** 2025-09-07

**Authors:** Fonny Cokro

**Affiliations:** Department of Clinical and Soc, Universitas Indonesia, Depok, West Java, Indonesia

Dear Dr. Aimen Shafiq,

 Thank you very much for your feedback.

 1) We have revised the sentences accordingly.

 2) We have revised the sentences accordingly.

 3) Patients were not randomized in this retrospective cohort study; however, all patients with a history of using dapagliflozin or empagliflozin for at least 12 months were selected, as outlined in the Methods section.

 Best regards,

 Authors

# Reviewer report: Reviewer response for version 1

**DOI:** 10.5256/f1000research.180344.r401370

**Published:** 2025-08-14

**Authors:** FARAZUL HODA

**Affiliations:** 1 Jamia Hamdard University, New Delhi, India

Research have been presented well but few of the comments from my end that will help authors to be present for fulfilment of the research presented. 

 1. Conclusion can be more elaborated write about the main findings and conclude appropriately.

 2. Method section can be more elaborated in study design and data sources. Explain about how data were collected and extracted, how they have screen the data, if any data in sheet is missing how they have handled.

 3. Better to add a follow diagram of participants selection and divide it into two groups for better presentation.

Is the work clearly and accurately presented and does it cite the current literature?

Yes

If applicable, is the statistical analysis and its interpretation appropriate?

Yes

Are all the source data underlying the results available to ensure full reproducibility?

Yes

Is the study design appropriate and is the work technically sound?

Partly

Are the conclusions drawn adequately supported by the results?

Partly

Are sufficient details of methods and analysis provided to allow replication by others?

Partly

Reviewer Expertise:

Diabetes and Diabetic Kidney Disease

I confirm that I have read this submission and believe that I have an appropriate level of expertise to confirm that it is of an acceptable scientific standard, however I have significant reservations, as outlined above.

# Response

**Published:** 2025-08-15

**Authors:** Fonny Cokro

**Affiliations:** Department of Clinical and Soc, Universitas Indonesia, Depok, West Java, Indonesia

Dear Dr. Farazul Hoda,

 Your feedback is greatly appreciated. We have made the following revisions to this article in response to your feedback. We hope our revisions meet your expectations. Our summary response is as follows:

- **Recommendation:** Conclusion can be more elaborated write about the main findings and conclude appropriately. **Response:** Thank you for your valuable feedback. We have revised the conclusion to meet your recommendations, which elaborates more about the findings.

- **Recommendation:** Method section can be more elaborated in study design and data sources. Explain about how data were collected and extracted, how they have screen the data, if any data in sheet is missing how they have handled. **Response:** Thank you for your valuable feedback. We have added explanations about how data were collected and extracted in “Study Design and Data Sources”. We had provided information about missing data handling in the “Variables” section.

- **Recommendation:** Better to add a follow diagram of participants selection and divide it into two groups for better presentation. **Response: **Thank you for your valuable feedback. We have revised the flow diagram to meet your recommendations, as follows: 1) We missed the arrow from the number of inclusion to the number of analyzed patients, thus we added the arrow; 2) We added the number of exclusions based on the reasoning to provide a better description; 3) We changed the diagram to make it more symmetrical and have a better presentation.

Best regards,

 Authors
